# Introduction to Special Thematic Issue, part 2 "Microsaccades: Empirical Research and Methodological Advances“

**DOI:** 10.16910/jemr.13.5.1

**Published:** 2023-03-25

**Authors:** Rudolf Groner

**Affiliations:** Journal of Eye Movement Research; University of Bern

**Keywords:** microsaccades, cognitive, sensory, pupillary, affective priming, visual search, torsion, main sequence, nystagmus, visual load, mental load

## Abstract

Microsaccades are at the interface between basic oculomotor phenomena and complex processes of cognitive functioning, and they also have been a challenge for subtle experimentation and adequate statistical analysis.

In the second part of the special thematic issue (for the first part see  4) the authors present a series of articles which demonstrate that microsaccades are still an interesting and rewarding area of scientific research the forefront of research in many areas of sensory, perceptual, and cognitive processes..

In their article “Pupillary and microsaccadic responses to cognitive effort and emotional arousal during complex decision making” Krejtz, Żurawska, Duchowski, & Wichary (1) investigate pupillary and microsaccadic responses to information processing during multi-attribute decision making under affective priming. The participants were randomly assigned into three affective priming conditions (neutral, aversive, and erotic) and instructed to make discriminative decisions. As hypothesized by the authors, the results showed microsaccadic rate inhibition and pupillary dilation, depending on cognitive effort prior to decision and moderated by affective priming. Aversive priming increased pupillary and microsaccadic responses to information processing effort. The results indicate that pupillary response is more influenced by affective priming than microsaccadic rate. The results are discussed in the light of neuropsychological mechanisms of pupillary and microsaccadic behavior.

In the article “Microsaccadic rate signatures correlate under monocular and binocular stimulation conditions” Essig, Leube, Rifai, & Wahl (2020) investigate microsaccades with respect to their directional distribution and rate under monocular and binocular conditions. In both stimulation conditions participants fixated a Gabor patch presented randomly in orientation of 45° or 135° over a wide range of spatial frequencies. Microsaccades were mostly horizontally oriented regardless of the spatial frequency of the grating. This outcome was consistent between both stimulation conditions. This study found that the microsaccadic rate signature curve correlates between both stimulation conditions, therefore extending the use of microsaccades to clinical applications, since parameters as contrast sensitivity, have frequently been measured monocularly in the clinical studies.

The study “Microsaccades during high speed continuous visual search” by Martin, Davis, Riesenhuber, & Thorpe (3) provides an analysis of the microsaccades occurring during visual search, targeting to small faces pasted either into cluttered background photos or into a simple gray background.  Participants were instructed to target singular 3-degree upright or inverted faces in changing scenes.  As soon as the participant’s gaze reached the target face, a new face was displayed in a different random location.  Regardless of the experimental context (e.g. background scene, no background scene), or target eccentricity (from 4 to 20 degrees of visual angle), The authors found that the microsaccade rate dropped to near zero levels within 12 milliseconds. There were almost never any microsaccades after stimulus onset and before the first saccade to the face. In about 20% of the trials, there was a single microsaccade that occurred almost immediately after the preceding saccade’s offset.  The authors argue that a single feedforward pass through the visual hierarchy of processing a stimulus is needed to effectuate prolonged continuous visual search and provide evidence that microsaccades can serve perceptual functions like correcting saccades or effectuating task-oriented goals during continuous visual search.

While many studies have characterized the eye movements during visual fixation, including microsaccades, in most cases only horizontal and vertical components have been recorded and analyzed. Little is known about the torsional component of microsaccades. In the study “Torsional component of microsaccades during fixation and quick phases during optokinetic stimulation” Sadeghpour & Otero-Millan (5) recorded eye movements around the three axes of rotation during fixation and torsional optokinetic stimulus. The authors found that the average amplitude of the torsional component of microsaccades during fixation was 0.34 ± 0.07 degrees with velocities following a main sequence with a slope comparable to the horizontal and vertical components. The size of the torsional displacement during microsaccades was correlated with the horizontal but not the vertical component. In the presence of an optokinetic stimulus a nystagmus was induced producing  more frequent and larger torsional quick phases compared to microsaccades produced during fixation of a stationary stimulus. The torsional component and the vertical vergence component of quick phases increased with higher velocities.

In previous research, microsaccades have been interpreted as psychophysiological indicators of task load. So far, it is still under debate how different types of task demands are influencing microsaccade rate. In their article “The interplay between task difficulty and microsaccade rate: Evidence for the critical role of visual load“ Schneider et al. (6) examined the relation between visual load, mental load and microsaccade rate. The participants carried out a continuous performance task (n-back) in which visual task load (letters vs. abstract figures) and mental task load (1-back to 4-back) were manipulated as within-subjects variables. Eye tracking data, performance data as well as subjective workload were recorded. Data analysis revealed an increased level of microsaccade rate for stimuli of high visual demand (i.e. abstract figures), while mental demand (n-back-level) did not modulate microsaccade rate. The authors concluded that microsaccade rate reflects visual load of a task rather than its mental load. This conclusion is in accordance with the proposition of Krueger et al. (2) “Microsaccades distinguish looking from seeing”, linking sensory with cognitive phenomena.

The present special thematic issue adds several new interesting facets to the research landscape around microsaccades. They still remain an attractive focus of interdisciplinary research and transdisciplinary applications. Thus, as already noted in the first part of this special thematic issue, research on microsaccades will not only endure, but keep evolving as the knowledge base expands.
